# Obituary

**Published:** 1888-05

**Authors:** 


### EDITORIAL.

### Obituary.

The head of the veteran editor of the REGISTER is bowed in grief.

The partner of all his joys and all his sorrows, for more than forty-six years, was suddenly called to her eternal home without a moments warning and without the melancholy pleasure of bidding adieu to the dear ones she loved so well.

Hannah Collins Taft, the wife of our own Dr. J. Taft.—She had been visiting her son and upon returning to her home at Riverside on Saturday, April 14th, met her death in sight of her own house and but a few steps away. She alighted from the street car at the small foot crossing opposite her home, which crosses the Big Four and O. & M. Railroad tracks. Upon reaching the crossing a freight train on the O. & M. road came rushing up and though the engineer sounded the warning, it came too late, for, in her bewilderment she attempted to cross the track and in doing so was struck by the engine and death was instantaneous.

So ended, in the first blush of Spring, a long and beautiful life —one devoted to God, her family and christian charity.

The funeral services were held at the homestead "On the Banks of the Beautiful River," amidst the tears of the bereaved family and a host of sympathizing friends.

Such a womanly character as this finds its way into the hearts of all who are fortunate enough to meet its possessor, and leaves behind it an impress of love and affection for human kind that enobles the mind.

This found its expression in the offer of sweet flowers—the lilies of the field, mute, but more eloquent than the poet's pen.

Truly did she exemplify the admonition of the immortal bard:

So live, that when thy summons comes to join
The innumerable caravan, which moves
To that mysterious realm, where each shall take
His chamber in the silent halls of death,
Thou go not, like the quarry-slave at night,
Scourged to his dungeon; but, sustained and soothed
By an unfaltering trust, approach thy grave,
Like one who wraps the drapery of his couch
About him, and lies down to pleasant dreams.

